# Supplementary material for: convertibleCARs: A chimeric antigen receptor system for flexible control of activity and antigen targeting
Source: Commun Biol. 2020 Jun 9;3:296. doi: 10.1038/s42003-020-1021-2 (PMC7283332; doi:10.1038/s42003-020-1021-2)
Supplement: Supplementary file 1 — Supplementary Information [file 42003_2020_1021_MOESM1_ESM.pdf]

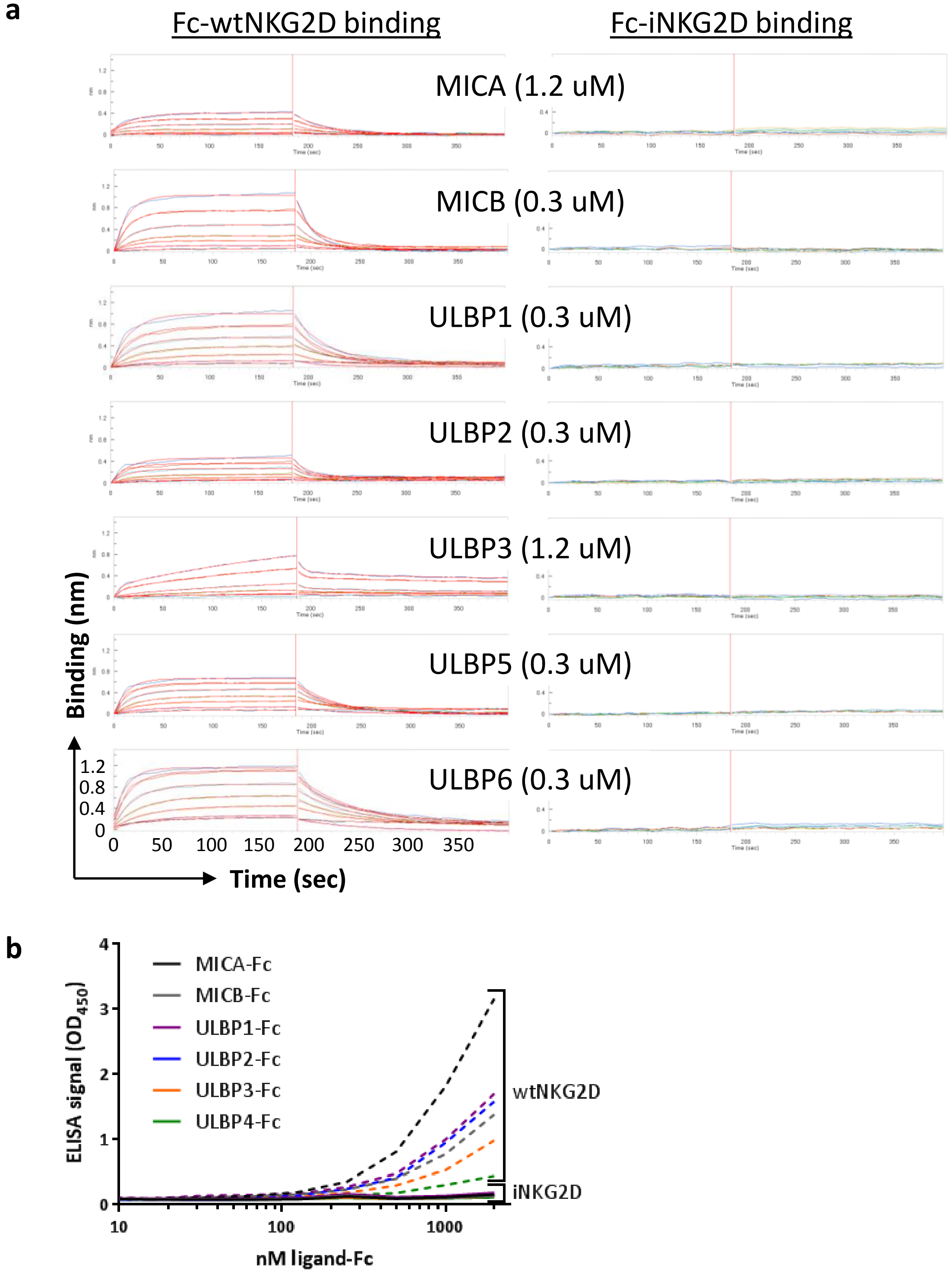

Supplementary Figure 1: (a) Octet BLI kinetic binding data for His-tagged monomeric wild-type MIC ligand interaction with either wild-type NKG2D or iNKG2D.YA. Fc-wtNKG2D or Fc-iNKG2D.YA were captured with anti-human IgG Fc capture (AHC) biosensor tips associated with a dilution series of each ligand (parenthetical value indicates highest concentration examined) after baseline establishment. ULBP4 could not be expressed and purified as a monomer so was not included in this assay. Note that all axes are to the same scale. Data is representative of a single experiment. **(b)** ELISA confirming inability of iNKG2D.YA to engage natural ligands. Ligand-Fc fusions (R&D Biosystems) were coated onto microtiter plates and a titration of biotinylated Fc-wtNKG2D (dashed lines) or Fc-iNKG2D.YA (solid lines) applied and detected by streptavidin-HRP.

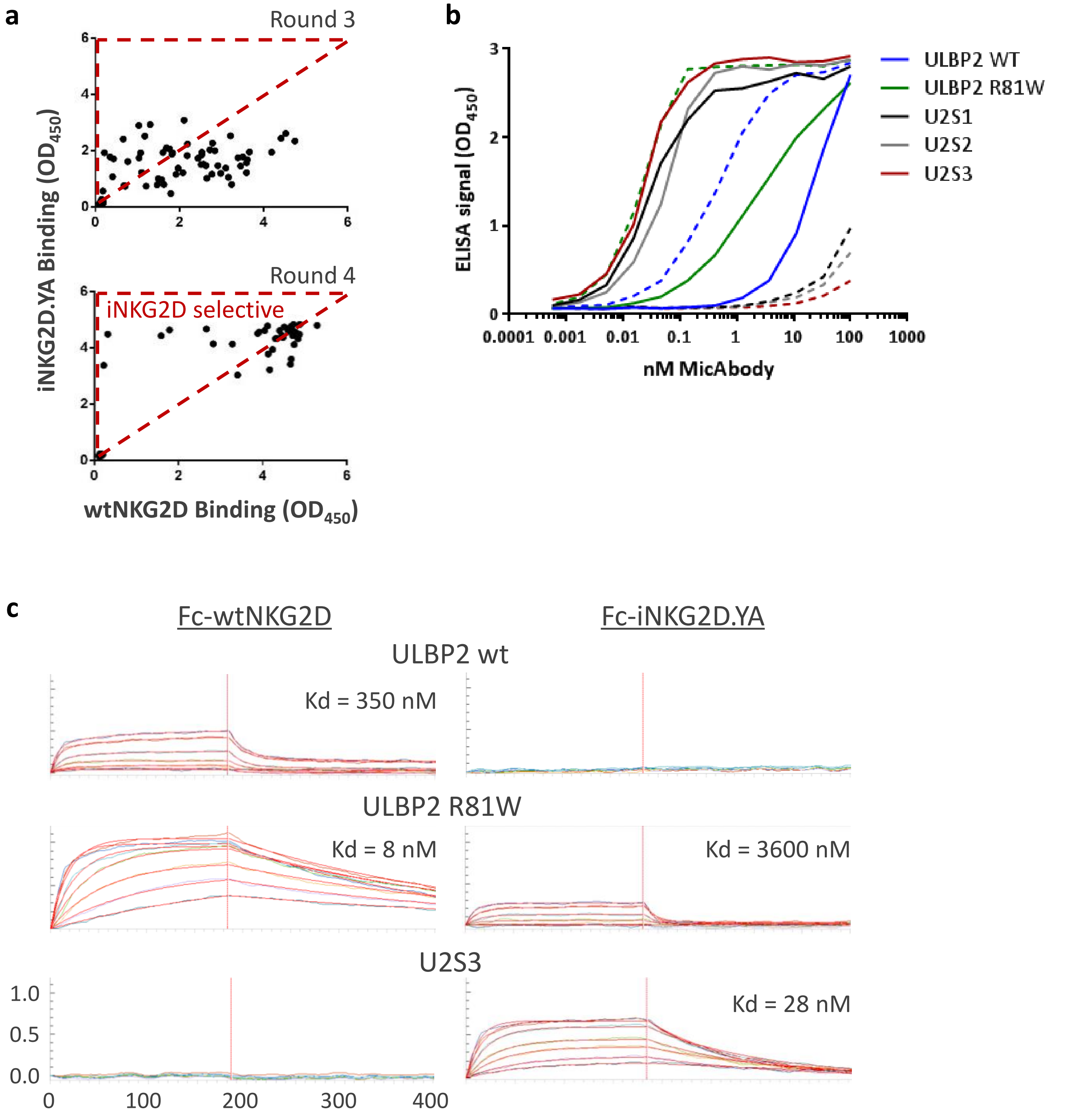

Supplementary Figure 2: (a) Relative binding of selected phage to Fc-iNKG2D.YA and Fc-wtNKG2D after the third and fourth rounds of panning in the presence of increasing concentrations of wtNKG2D competitor. Phage clones in the portion of the graph outlined by the red triangle were selected for further characterization. **(b)** Three phage variants – S1, S2, S3 – were expressed as fusions to the C-terminus of the anti-FGFR3 antibody clone R3Mab heavy chain as MicAbodies and, along with wild-type ULBP2 and R81W versions, were tested for the ability of the selective variants to retain preferential Fc-iNKG2D.YA binding (solid lines) over Fc-wtNKG2D (dashed lines). All purified MicAbodies retained binding to human FGFR3 (data not shown). **(c)** Binding analysis of His-tagged monomeric wild-type ULBP2, ULBP2 R81W, and the orthogonal U2S3 ligand binding to Fc-NKG2D and Fc-iNKG2D.YA. Fc-wtNKG2D or Fc-iNKG2D.YA were captured with anti-human IgG Fc capture (AHC) biosensor tips then associated with a dilution series of ligand. Data are from single experiments.

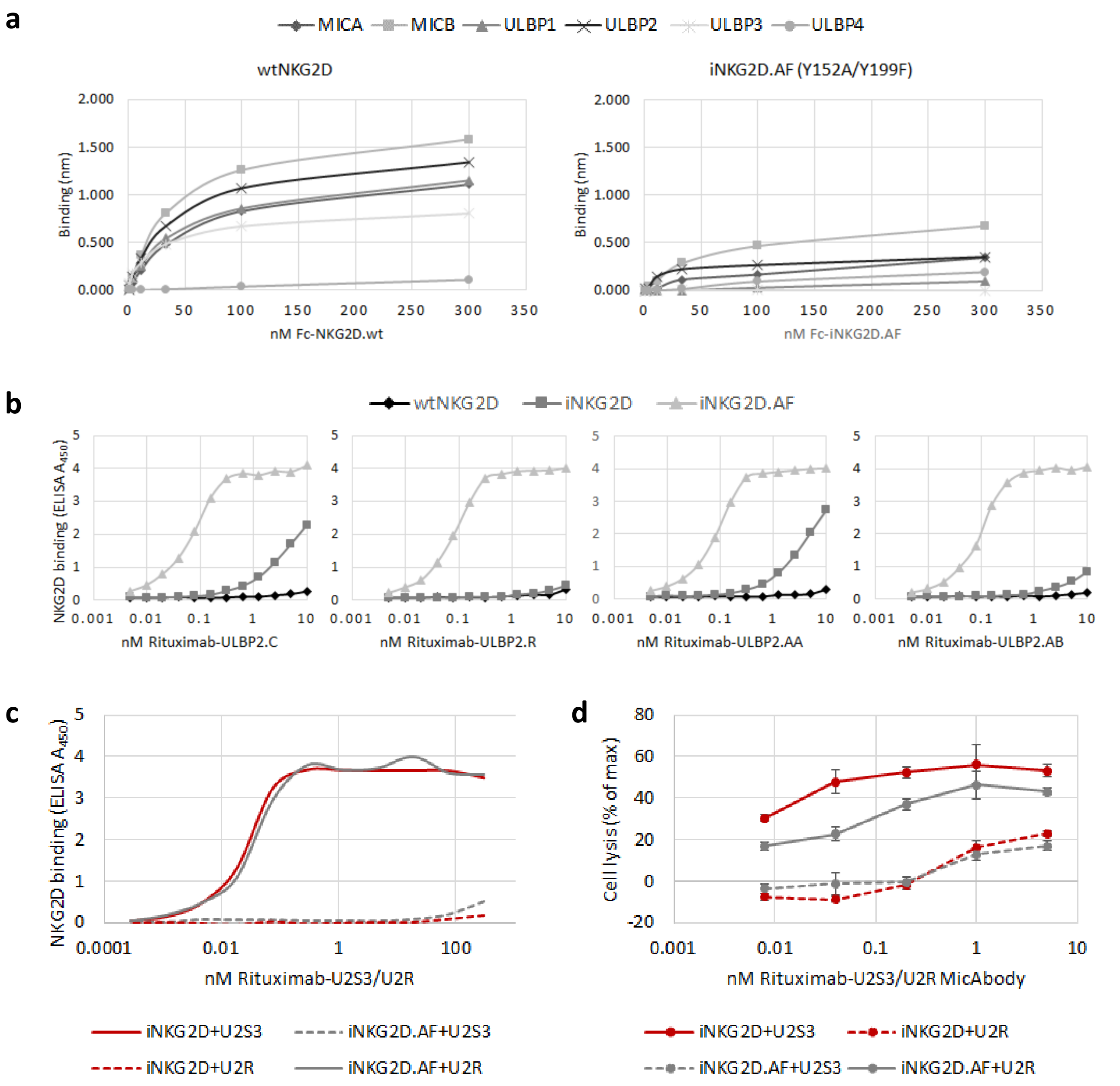

Supplementary Figure 3: Orthogonal ULBP2 ligand selective binding to NKG2D Y152A/199F (iNKG2D.AF). Library design and phage panning performed as described for iNKG2D.YA except that biotinylated double-mutant Fc-iNKG2D.AF was used during rounds of selection against increasing concentrations of Fc-wtNKG2D competitor. Data represents a single experiment **(a)** Octet BLI binding data for interaction of monomeric ligands to either Fc-wtNKG2D or Fc-iNKG2D.AF. Data are representative of two experiments **(b)** Lead variants selected from the phage display library were cloned as fusions to the C-terminus of the Rituximab light chain and differential binding to Fc-wtNKG2D, Fc-iNKG2D.YA, and Fc-iNKG2D.AF and quantified by ELISA. Shown are four variants that selectively engage Fc-iNKG2D.YA and not the other two receptors. **(c)** ELISA demonstrating exclusivity of U2S3 and U2R ligand binding to the receptor variant against which it was selected – Fc-iNKG2D.YA and Fc-iNKG2D.AF, respectively. **(d)** Calcein release assay with Ramos target cells at an E:T of 20:1 with either iNKG2D.YA-CAR or iNKG2D.AF-CAR expressing CD8+ T cells and a titration of Rituximab.LC-U2S3 or Rituximab.LC-U2R. Error bars represent ±SD of technical replicates.

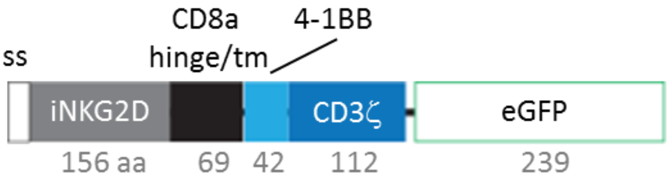

<u>MALPVTALLLPLALLLHAARP</u>LFNQEVQIPLTESYCGPCPKNWICYKNNCYQFFDESKNWYESQASCMS
QNASLLKVYSKEDQDLLKLVKSAHWMGLVHIPTNGSWQWEDGSILSPNLLTIIEMQKGDCALYASSFKG
FIENCSTPNTYICMQRTVTTTPAPRPPTPAPTIASQPLSLRPEACRPAAGGAVHTRGLDFACDIYIWAPLA
GTCGVLLLSLVITLYCSLKRGRKKLLYIFKQPFMRPVQTTQEEDGCSCRFPEEEEGGCELRVKFSRSADAPAY
KQGQNQLYNELNLGRREEYDVLDKRRGRDPEMGGKPRRKNPQEGLYNELQKDKMAEAYSEIGMKGER
RRGKGHDGLYQGLSTATKDTYDALHMQALPPRSGSGSGSGSMVSKGEELFTGVVPILVELDGDVNGHK
FSVSGEGEGDATYGKLTLKFICTTGKLPVPWPTLVTTLTYGVQCFSRYPDHMKQHDFFKSAMPEGYVQE
RTIFFKDDGNYKTRAEVKFEGDTLVNRIELKGIDFKEDGNILGHKLEYNYNSHNVYIMADKQKNGIKANF
KIRHNIEDGSVQLADHYQQNTPIGDGPVLLPDNHYLSTQSALSKDPNEKRDHMVLLEFVTAAGITLGMD
ELYK

Supplementary Figure 4: Schematic of the iNKG2D.YA CAR receptor starting from the N-terminus of the polypeptide on the left and includes the signal sequence (SS) which is absent in the mature type I transmembrane protein. Domains are color-coded in the sequence to match the diagram.

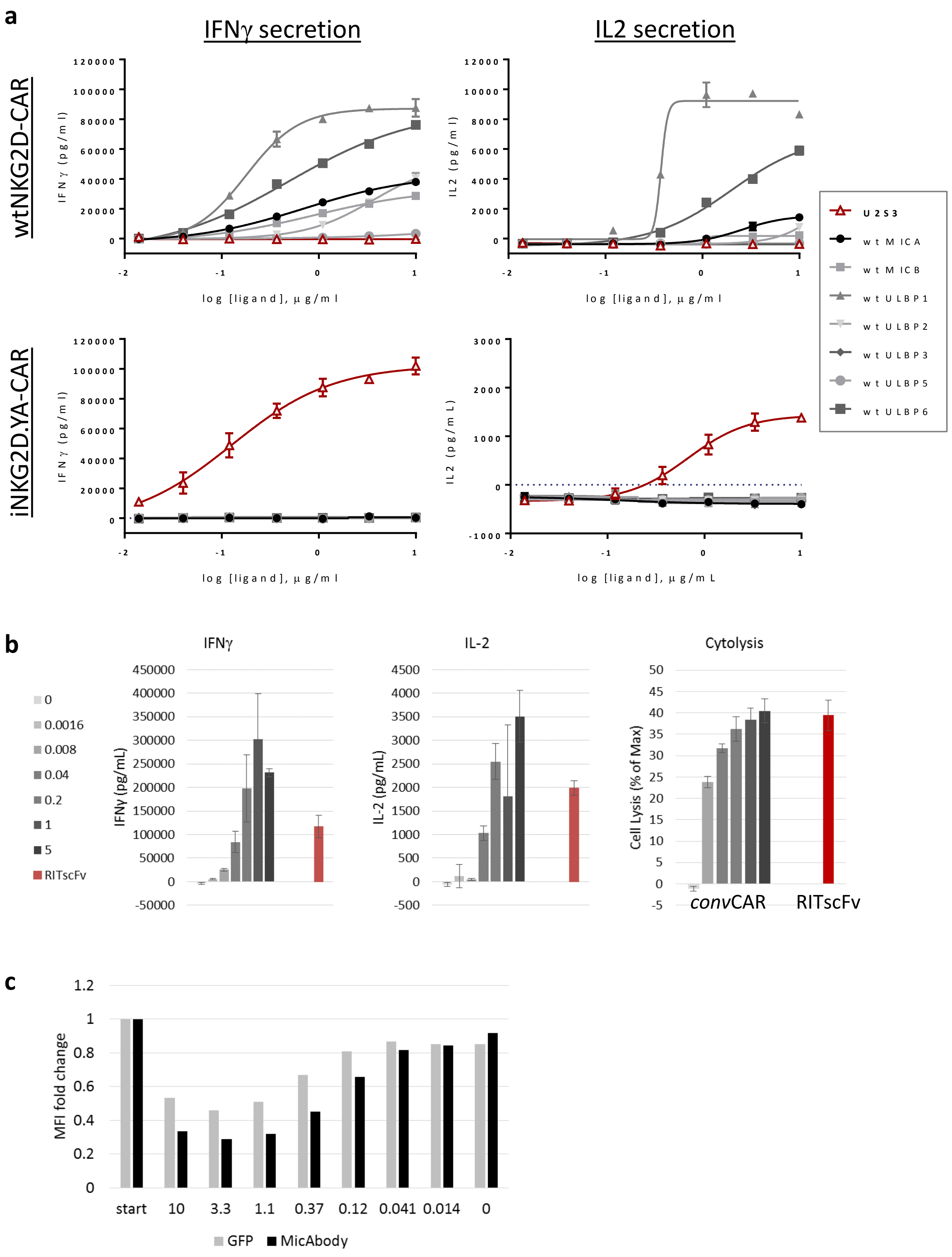
a
IFNγ secretion
IL2 secretion
wtNKG2D-CAR
iNKG2D.YA-CAR
IFNγ (pg/ml)
IL2 (pg/ml)
IL2 (pg/mL)
log [ligand], µg/ml
log [ligand], µg/mL
120000
100000
80000
60000
40000
20000
0
12000
10000
8000
6000
4000
2000
3000
1000
-1000
-2
-1
1
U2S3
wtMICA
wtMICB
wtULBP1
wtULBP2
wtULBP3
wtULBP5
wtULBP6
b
IFNγ
IL-2
Cytolysis
0
0.0016
0.008
0.04
0.2
1
5
RITscFv
IFNγ (pg/mL)
450000
400000
350000
300000
250000
200000
150000
100000
50000
-50000
IL-2 (pg/mL)
4500
4000
3500
3000
2500
2000
1500
1000
500
-500
Cell Lysis (% of Max)
50
45
40
35
30
25
20
15
10
5
-5
convCAR
RITscFv
c
MFI fold change
1.2
0.8
0.6
0.4
0.2
start
10
3.3
1.1
0.37
0.12
0.041
0.014
GFP
MicAbody

Supplementary Figure 5: Ligand-dependent activation of iNKG2D-CAR expressing $CD8^+$ T cells and MicAbody-dependent receptor internalization. **(a)** CD8+ T cells were transduced with CAR constructs comprised of either wild-type NKG2D or iNKG2D.YA as the receptor domain. Wild-type His-tagged monomeric ligands or His-tagged monomeric U2S3 were coated onto the wells of a microtiter plate in a 1:3 dilution series starting at 10 ug/mL. $1x10^5$ CAR expressing cells were introduced to the wells in 150 uL volume without exogenous IL2, supernatants collected 24 hours later, and the amount of cytokine produced and release quantified by cytokine-specific ELISA. ULBP4 was not included in the assay as a His-tagged version could not be expressed and purified. **(b)** CD8+ cells expressing either iNKG2D-CAR or RITscFv-CAR were co-cultured with Ramos cells at an E:T of 4:1 with increasing concentrations of Rit-S3 MicAbody (nM) in the case of iNKG2D-CAR cells. After 24 hours, culture supernatants were harvested and released cytokine quantified by ELISA. Cytolysis was measured by calcein release after two hours of co-incubation. All error bars are $\pm$SD of technical triplicates. All data representative of a single experiment. **(c)** iNKG2D-$CD8^+$ cells were pre-incubated 5 nM Trastuzumab.LC-U2S3 MicAbody then exposed to wells pre-coated with a titration of Her2. After 2 hours, cells were incubated with anti-kappa-PE antibody to detect surface accessible MicAbody and GFP was examined to look for total levels of expressed iNKG2D-CAR.

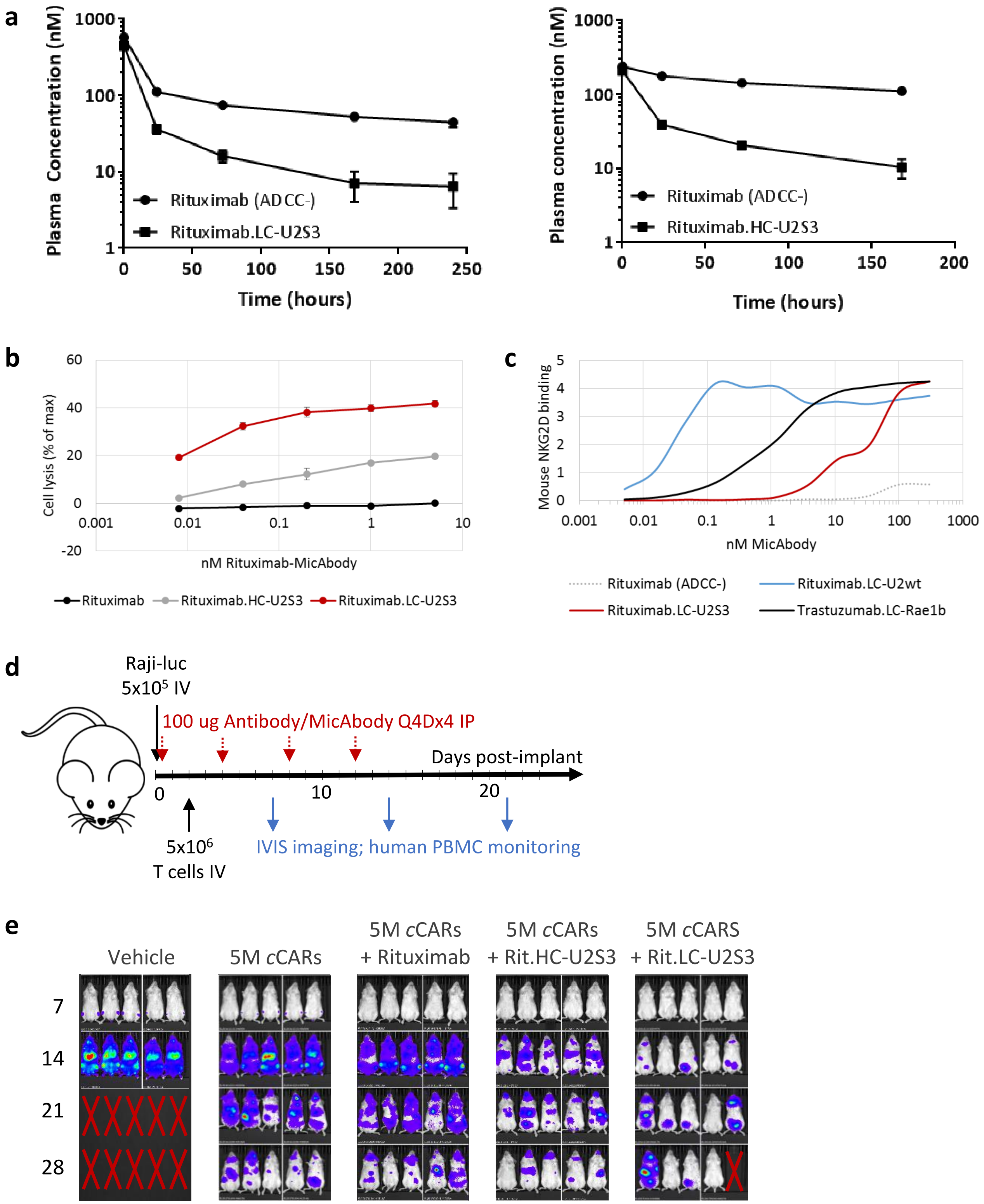

a
Plasma Concentration (nM)
Time (hours)
Rituximab (ADCC-)
Rituximab.LC-U2S3
Plasma concentration (nM)
Time (hours)
Rituximab (ADCC-)
Rituximab.HC-U2S3
b
Cell lysis (% of max)
nM Rituximab-MicAbody
Rituximab
Rituximab.HC-U2S3
Rituximab.LC-U2S3
c
Mouse NKG2D binding
nM MicAbody
Rituximab (ADCC-)
Rituximab.LC-U2wt
Rituximab.LC-U2S3
Trastuzumab.LC-Rae1b
d
Raji-luc
5x10⁵ IV
100 ug Antibody/MicAbody Q4Dx4 IP
Days post-implant
5x10⁶
T cells IV
IVIS imaging; human PBMC monitoring
e
Vehicle
5M cCARs
5M cCARs + Rituximab
5M cCARs + Rit.HC-U2S3
5M cCARS + Rit.LC-U2S3
7
14
21
28

Supplementary Figure 6: Comparison of heavy- vs. light-chain U2S3 fusions to Rituximab (ADCC-) antibody. **(a)** Pharmacokinetics of serum Rituximab-U2S3 MicAbody levels after 100 ug IV administration in NSG mice in the absence of human T cells or tumor. Note that all MicAbodies and antibody controls used were ADCC-deficient. The graph on the left is a comparison of parental antibody to the light-chain U2S3 fusion while the graph on the right is a comparison of parental antibody to the heavy-chain U2S3 fusion. All error bars are ±SD of technical triplicates. **(b)** *In vitro* calcein release assay after two hours co-culture with iNKG2D-CAR CD8+ T cells and Ramos target cells at an E:T of 20:1 and titrations of Rituximab-MicAbodies. Error bars represent ±SD for the experiment and data are representative of multiple experiments. **(c)** ELISA demonstrating binding of Rituximab.LC-U2S3 to mouse NKG2D. Shown are the A480 absorbance values. Trastuzumab.LC-Rae1b, with a mouse wild-type Rae1b ligand that binds naturally to mouse NKG2D, was included as a positive control. **(d)** *In vivo* study design in NSG mice, n=5, with Raji-luciferase cells invused IV followed by treatment and monitoring as indicated. CD4 and CD8 cells were independently transduced, combined at a 1:1 ratio without adjusting for percent transduction, and a total of $5x10^6$ *convertible*CAR-T cells (*c*CARs) were injected IV. **(e)** IVIS imaging was performed 7, 14, 21, and 28 days post-implantation and all adjusted to the same scale. Death of mouse #5 at day 28 of the 5M iNKG2D + Rituximab.LC-U2S3 was acute and attributed to the bright focal mass located in the proximity of the brainstem that was observed at day 21.

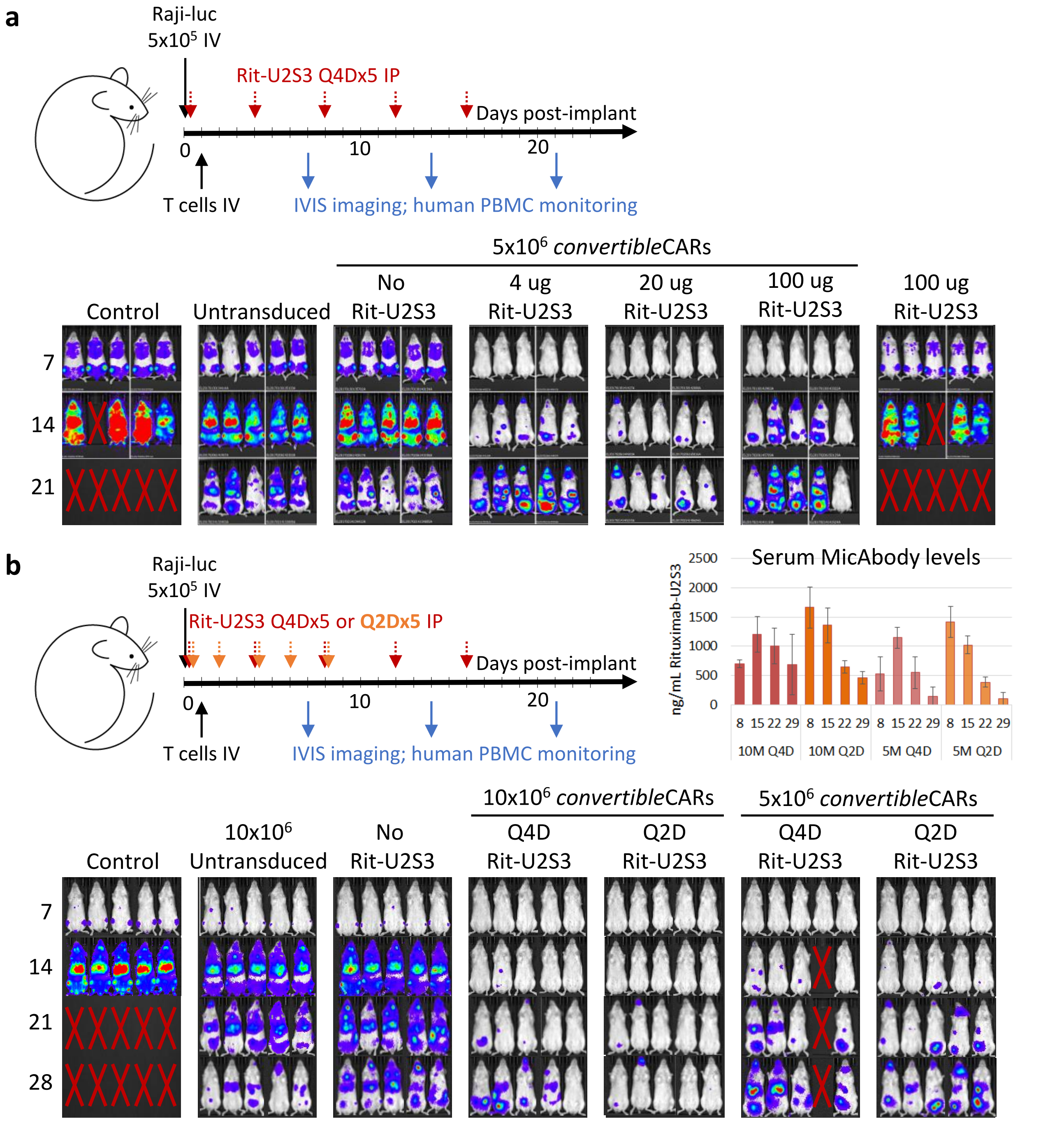

Supplementary Figure 7: Investigation of both MicAbody and *convertible*CAR dosing strategy in a disseminated Raji-luciferase B cell lymphoma NSG mouse model. **(a)** Rituximab.LC-U2S3 Q4Dx5 dosing was initiated the same day as tumor implantation at 4, 20, or 100 ug per dose while keeping the number of cells administered constant across cohorts. The 100 ug Rituximab.LC-U2S3 only cohort – without any CAR-T cells – received just a single dose of MicAbody. **(b)** Frequency of MicAbody dosing as well as *convertible*CAR-T cell infusions levels were explored with the former being administered either every two or four days for a total of 5 doses, and the latter at either $5x10^6$ or $10x10^6$ total cells infused. Mouse #4 in the Q4D + $5x10^6$ cohort that died by day 14 did so for reasons unrelated to treatment or disease. Serum levels of Rituximab.LC-U2S3 were also monitored at 8, 15, 22, and 29 days post-implantation for all cohorts that received MicAbody (cohorts that did not receive any were negative by ELISA). Error bars are ±SD across the samples of a given cohort. A non-CAR specific graft-vs-tumor effect was clearly observed in this study with the untransduced and no MicAbody cohorts. IVIS images within each respective study were adjusted to the same scale.

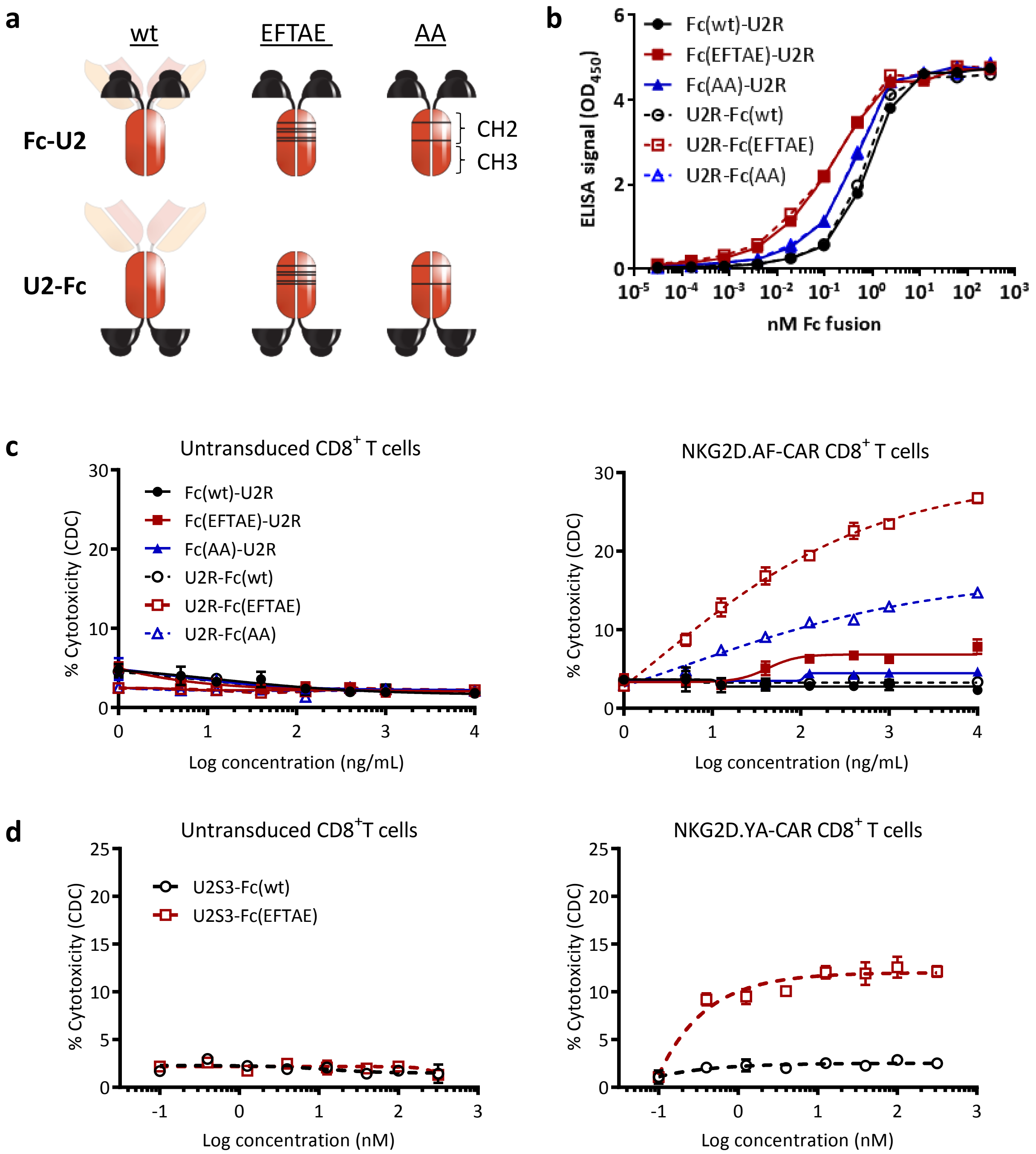
a
wt
EFTAE
AA
Fc-U2
U2-Fc
CH2
CH3
b
Fc(wt)-U2R
Fc(EFTAE)-U2R
Fc(AA)-U2R
U2R-Fc(wt)
U2R-Fc(EFTAE)
U2R-Fc(AA)
ELISA signal (OD450)
nM Fc fusion
c
Untransduced CD8+ T cells
NKG2D.AF-CAR CD8+ T cells
% Cytotoxicity (CDC)
Log concentration (ng/mL)
d
Untransduced CD8+T cells
NKG2D.YA-CAR CD8+ T cells
U2S3-Fc(wt)
U2S3-Fc(EFTAE)
% Cytotoxicity (CDC)
Log concentration (nM)

Supplementary Figure 8: Targeted recruitment of complement factor C1q to iNKG2D.AF-CAR cells to direct their complement-mediated attrition. **(a)** Structure of orthogonal ligand fusions to the Fc portion of human IgG expressed as either N- or C-terminal fusions. In addition to wild-type Fc, two sets of mutations in the CH2 domain that enhance C1q binding were independently explored - S267E/H268F/S324T/G236A/I332E ("EFTAE") and K326A/E333A ("AA"). **(b)** ELISA examining binding of human C1q to each purified fusion protein. Rank order of Kd's was EFTAE<AA<wt (0.12, 0.35, and 0.67 nM, respectively) regardless of orientation of fusions. **(c)** Complement-dependent cytotoxicity (CDC) assays for C1q-binding enhance Fc-fusions. iNKG2D.AF-CAR or untransduced $CD8^+$ T cells were incubated with a titration of each fusion molecule and 10% normal human serum complement for three hours before dead T cells were enumerated with SYTOX Red. **(d)** CDC assays with U2S3 orthogonal ligand fusions to direct a complement to iNKG2D.YA-CAR cells as described in (c) above. All error bars are $\pm$SD of triplicate technical measurements with The iNKG2D-AF and iNKG2D-YA performed as separate experiments.

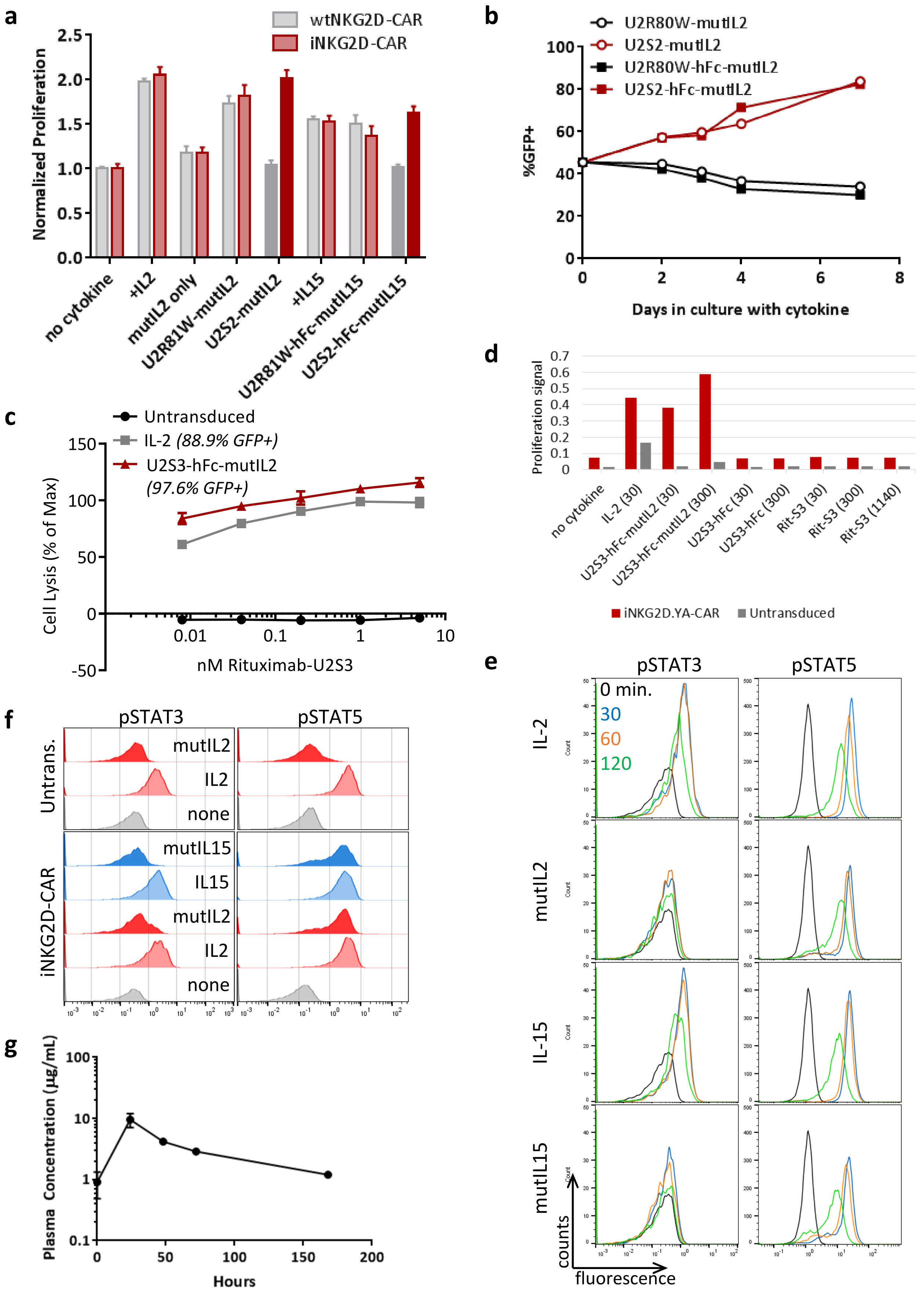
a
wtNKG2D-CAR
iNKG2D-CAR
Normalized Proliferation
no cytokine
+IL2
mutIL2 only
U2R81W-mutIL2
U2S2-mutIL2
+IL15
U2R81W-hFc-mutIL15
U2S2-hFc-mutIL15
b
U2R80W-mutIL2
U2S2-mutIL2
U2R80W-hFc-mutIL2
U2S2-hFc-mutIL2
%GFP+
Days in culture with cytokine
c
Untransduced
IL-2 (88.9% GFP+)
U2S3-hFc-mutIL2 (97.6% GFP+)
Cell Lysis (% of Max)
nM Rituximab-U2S3
d
Proliferation signal
no cytokine
IL-2 (30)
U2S3-hFc-mutIL2 (30)
U2S3-hFc-mutIL2 (300)
U2S3-hFc (30)
U2S3-hFc (300)
Rit-S3 (30)
Rit-S3 (300)
Rit-S3 (1140)
iNKG2D.YA-CAR
Untransduced
e
pSTAT3
pSTAT5
0 min.
30
60
120
IL-2
mutIL2
IL-15
mutIL15
counts
fluorescence
f
pSTAT3
pSTAT5
Untrans.
iNKG2D-CAR
mutIL2
IL2
none
mutIL15
IL15
g
Plasma Concentration (μg/mL)
Hours

Supplementary Figure 9: Targeted delivery of mutant-IL2 cytokine to iNKG2D-CAR CD8+ T cells. **(a)** *in vitro* proliferation after three days of wtNKG2D-CAR or iNKG2D.YA-CAR treatment with 30 IUe/mL of cytokine or cytokine-U2S2 fusion. Darker shading is to highlight selectivity. **(b)** A low efficiency (45% GFP^+) iNKG2D.YA-CAR transduction was cultured with 30 IUe/mL of non-selective (U2R81W) or iNKG2D.YA-selective (U2S2) mutIL2 fusion and maintained for seven days. Cells were periodically examined by flow cytometry to quantify the $\%GFP^+$ cells in each population. **(c)** iNKG2D-CAR $CD8^+$ T cells were cultured with 30 IUe/mL of either wild-type IL-2 or U2S3-hFc-mutIL2 then co-cultured with Ramos cells at an E:T of 20:1 with increasing concentrations of Rituximab.LC-U2S3. Liberated calcein was quantified and untransduced $CD8^+$ cells maintained in rhIL-2 served as a negative control. **(d)** Untransduced or iNKG2D-CAR CAR CD8+ T cells were incubated with various cytokine molecules for three-days and proliferation quantified. Control molecules included a monomeric U2S3-hFc as well as Rit-S3 MicAbody. Parenthetical values are IUe/mL concentrations tested. Data shown are an average of technical triplicates. **(e)** Untransduced or iNKG2D-CAR CD8+ T cells were starved overnight of supporting cytokine then treated with 150 IUe/mL IL-2, IL-15, U2S3-hFc-mutIL2, or U2S3-hFc-mutIL15 for two hours before fixing and staining for intracellular phospo-STAT3 and –STAT5. **(f)** iNKG2D-CAR CD8+ T cells were treated as in (d) except that cells were fixed at 0, 30, 60, and 120 minutes after exposure to cytokines or U2S3-hFc-cytokine fusions then stained for intracellular phospo-STAT3 and –STAT5. **(g)** Serum PK of U2S3-hFc-mutIL2 after 60 ug IP injection in NSG mice (N=3). All error bars are $\pm$SD of biological triplicates. With the exception of Supplementary Figure 9e and 9f, data are representative of at least two experiments.

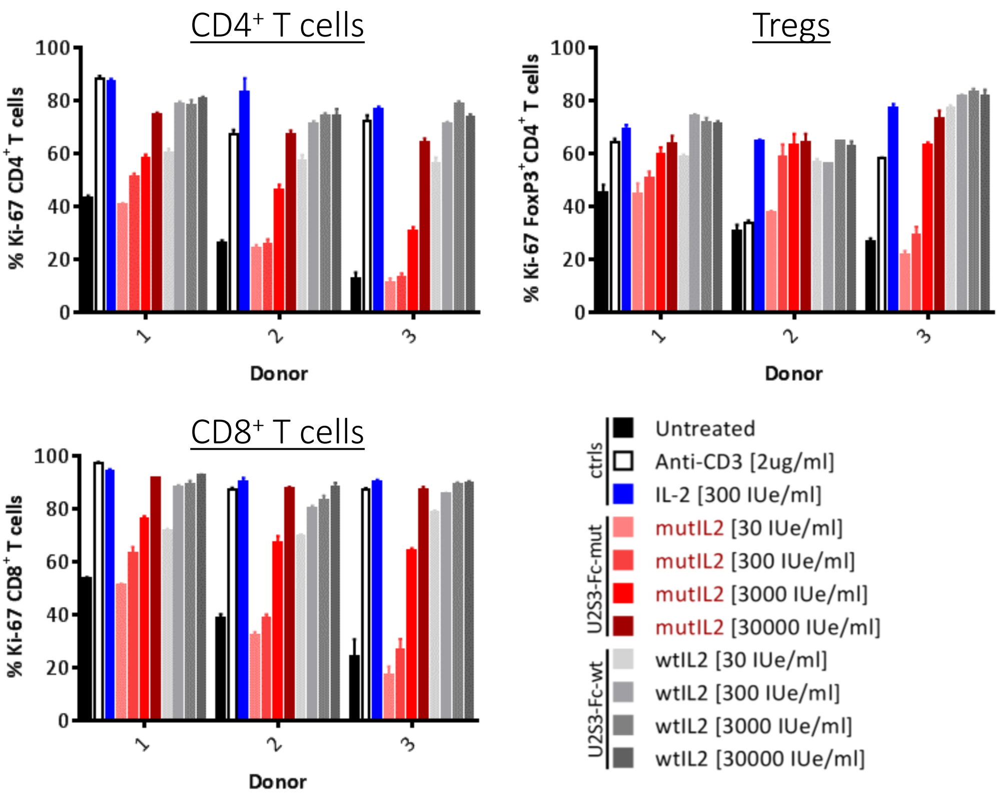

Supplementary Figure 10: Responsiveness of human PBMCs to U2S3-hFc-mutIL2. Human PBMCs from three donors were incubated with increasing concentrations of U2S3-hFc-mutIL2 or U2S3-hFc-wtIL2 for four days along with controls. Each of the labeled cell types was examined for the marker Ki-67 to quantify proliferative response under each condition. Error bars are ±SD of triplicate measurements and data represents a single experiment.

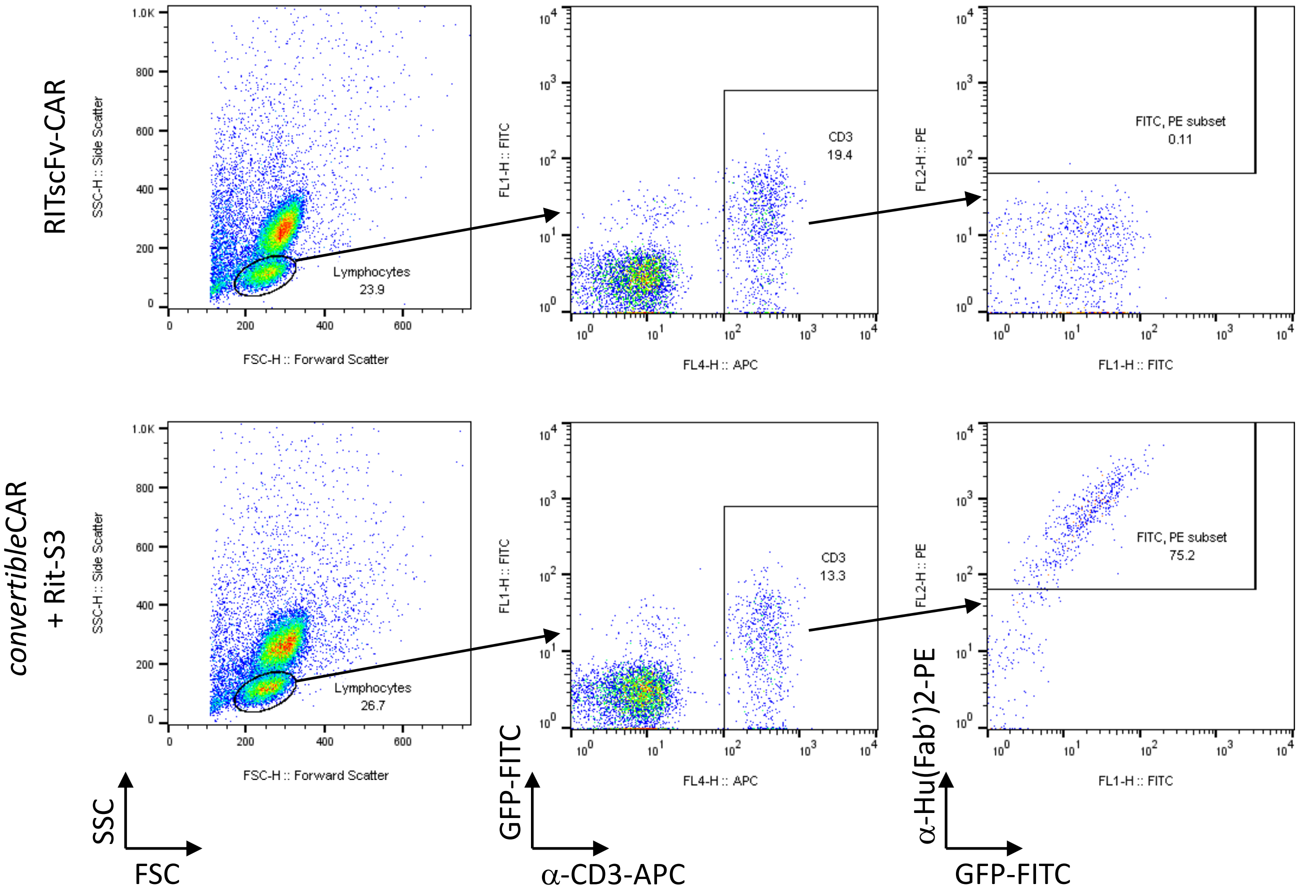

Supplementary Figure 11: Example flow cytometry gating scheme using a peripheral bloods samples from a mouse that received RITscFv-CARs and a mouse that received *convertible*CAR-T+Rit-S3.
