# Supplementary material for: convertibleCARs: A chimeric antigen receptor system for flexible control of activity and antigen targeting
Source: Commun Biol. 2020 Jun 9;3:296. doi: 10.1038/s42003-020-1021-2 (PMC7283332; doi:10.1038/s42003-020-1021-2)
Supplement: Supplementary file 2 — Description of Additional Supplementary Files [file 42003_2020_1021_MOESM2_ESM.pdf]

Description of Supplementary Data

Tab Name	Corresponding Figure	Description
Fig3A	Figure 3a	ELISA absorbance (A_{450}) data were interpolated into standard curves for IL-2 and IFN-gamma as appropriate and reported as pg/mL. Each co-incubation condition was set up in triplicate wells and each Excel cell represents the ELISA signal from one of those wells.
Fig3B	Figure 3b	MFI data as reported from our contract research organization and is representative of the average of replicate samples.
Fig3C	Figure 3c	Plate layout, raw data, and calculations for the calcein-release cytotoxicity assay.
Fig3D	Figure 3d	Plate layout, raw data, and calculations for the calcein-release cytotoxicity assay.
Fig4C_D_E	Figures 4c, 4d, 4e	Bioluminescent signal (total flux as p/s or photons/second) obtain from IVIS imaging of mice that had been implanted with luciferase-engineered Raji (Raji-luc) cells. Individual mouse measurements in each of the cohorts is shown as a function of time.
Fig4F	Figure 4f	%CD3 positive numbers obtained during flow cytometry analysis of mouse blood samples. Individual mouse values shown and arranged by cohort and time.
Fig4G	Figure 4g	% MicAbody surface staining as detected with the anti-Fab'2 antibody which binds the MicAbody. Reported here as a percentage of CD3+ve cells. Individual mouse values shown arranged by cohort and time.
Fig5B	Figure 5b	Tumor volumes reported in mm3 based upon caliper measurements using the formula length x width x width x 0.5. Shown are tumor volumes for each individual mouse arranged by cohort and time.
Fig5C	Figure 5c	Terminal tumor masses in grams for each mouse, organized by cohort.
Fig5D	Figure 5d	Serum MicAbody levels reported in ng/mL from an ELISA analysis were absorbance (A_{450}) values were interpolated into a standard curve. Shown are the values for individual mice arranged by cohort and time.
Fig5E	Figure 5e	Flow cytometry results for %CD3+ve and %MicAbody (Fab'2) positive cells in the blood of mice. Shown are values for individual mice arranged by cohort and time.
Fig6A	Figure 6a	%CD3 positive numbers obtained during flow cytometry analysis of mouse blood samples. Individual mouse values shown and arranged by cohort and time.
Fig6B	Figure 6b	%CD8 of CD3+ve cells and %GFP of CD8+/CD3+ numbers obtained during flow cytometry analysis of mouse blood samples. Individual mouse values shown and arranged by cohort and time.
