# Supplementary material for: convertibleCARs: A chimeric antigen receptor system for flexible control of activity and antigen targeting
Source: Commun Biol. 2020 Jun 9;3:296. doi: 10.1038/s42003-020-1021-2 (PMC7283332; doi:10.1038/s42003-020-1021-2)
Supplement: Supplementary file 4 — Reporting Summary [file 42003_2020_1021_MOESM4_ESM.pdf]

nature research

Corresponding author(s): Kaman Kim

Last updated by author(s): May 19, 2020

Reporting Summary

Nature Research wishes to improve the reproducibility of the work that we publish. This form provides structure for consistency and transparency in reporting. For further information on Nature Research policies, see Authors & Referees and the Editorial Policy Checklist.

Statistics

For all statistical analyses, confirm that the following items are present in the figure legend, table legend, main text, or Methods section.

n/a	Confirmed	
☐	☒	The exact sample size (n) for each experimental group/condition, given as a discrete number and unit of measurement
☐	☒	A statement on whether measurements were taken from distinct samples or whether the same sample was measured repeatedly
☐	☒	The statistical test(s) used AND whether they are one- or two-sided<br>*Only common tests should be described solely by name; describe more complex techniques in the Methods section.*
☒	☐	A description of all covariates tested
☒	☐	A description of any assumptions or corrections, such as tests of normality and adjustment for multiple comparisons
☐	☒	A full description of the statistical parameters including central tendency (e.g. means) or other basic estimates (e.g. regression coefficient) AND variation (e.g. standard deviation) or associated estimates of uncertainty (e.g. confidence intervals)
☐	☒	For null hypothesis testing, the test statistic (e.g. F, t, r) with confidence intervals, effect sizes, degrees of freedom and P value noted<br>*Give P values as exact values whenever suitable.*
☒	☐	For Bayesian analysis, information on the choice of priors and Markov chain Monte Carlo settings
☒	☐	For hierarchical and complex designs, identification of the appropriate level for tests and full reporting of outcomes
☒	☐	Estimates of effect sizes (e.g. Cohen's d, Pearson's r), indicating how they were calculated

Our web collection on statistics for biologists contains articles on many of the points above.

Software and code

Policy information about availability of computer code

Data collection	No software was used
Data analysis	No software was used

For manuscripts utilizing custom algorithms or software that are central to the research but not yet described in published literature, software must be made available to editors/reviewers. We strongly encourage code deposition in a community repository (e.g. GitHub). See the Nature Research guidelines for submitting code & software for further information.

Data

Policy information about availability of data

All manuscripts must include a data availability statement. This statement should provide the following information, where applicable:

- Accession codes, unique identifiers, or web links for publicly available datasets
- A list of figures that have associated raw data
- A description of any restrictions on data availability

Uniprot accession codes for all wild-type proteins incorporated into this study were provided in the Methods section. Raw data files have been provided for all of the charts and graphs incorporated in the main manuscript and can be accessed via the SupplementaryDataFile1.xlsx.

Field-specific reporting

Please select the one below that is the best fit for your research. If you are not sure, read the appropriate sections before making your selection.

☒ Life sciences ☐ Behavioural & social sciences ☐ Ecological, evolutionary & environmental sciences

Life sciences study design

All studies must disclose on these points even when the disclosure is negative.

Sample size	No a priori sample size calculation was performed and the selection of N=5 per cohort was primarily based upon precedent of other published studies for establishing proof of concept. Retrospective application of a crude calculation referred to as the "resource equation" method (PMID 24250214) indicates that the sample sizes used were sufficient.
Data exclusions	No data were excluded. Study results shown represent all the mice from the cohort as defined at the onset of the study.
Replication	Animal studies were not exactly replicated, but each study conducted informed on subsequent studies. As a consequence, every effort was made to include critical controls in every study (e.g. untransduced T cell cohort, no treatment cohorts).
Randomization	No randomization for disseminated studies was performed although all mice were given the same inoculum of tumor cells. For the subcutaneous model, outliers were discarded and mice redistributed prior to initiation of therapy such that the average tumor size was comparable between cohorts.
Blinding	The authors of the manuscript were never involved in execution of the study, only in the original design of study groups and treatment regimens then examination of the data as it was generated. All animal handling for efficacy studies, administration of test articles, sample extraction, and flow cytometry was performed by ProMab.

Reporting for specific materials, systems and methods

We require information from authors about some types of materials, experimental systems and methods used in many studies. Here, indicate whether each material, system or method listed is relevant to your study. If you are not sure if a list item applies to your research, read the appropriate section before selecting a response.

Materials & experimental systems

n/a	Involved in the study
☐	☒ Antibodies
☐	☒ Eukaryotic cell lines
☒	☐ Palaeontology
☐	☒ Animals and other organisms
☒	☐ Human research participants
☒	☐ Clinical data

Methods

n/a	Involved in the study
☒	☐ ChIP-seq
☐	☒ Flow cytometry
☒	☐ MRI-based neuroimaging

Antibodies

Antibodies used	PE-anti-human kappa chain (Abcam #ab79113), APC Anti-Human CD3 (clone OKT3, 20-0037-T100, Tonbo Biosciences), biotinylated Anti-Human F(ab')2 (109-066-097, Jackson ImmunoResearch Laboratories Inc.) with Streptavidin-PE detection (BD 554061), phospo-STAT3 (Biolegend PE anti-STAT3 Tyr705 clone 13A3-1), phospho–STAT5 (BD Alexa Fluor 647 anti-STAT5 pY694 clone 47), BioLegend antibody panels for proliferating T cells (CD8 clone RPA-T8 #301050, CD4 clone OKT4 #317410, CD3 clone OKT3 #300430, KI-67 #350514), Biolegend antibody panel for Treg cells (Fox3 clone 206D #320106, CD4 clone OKT4, CD3 clone OKT3, KI-67).
Validation	Validation statements provided by the manufacturer for each product were the primary resource for specificity of reactivity.

Eukaryotic cell lines

Policy information about cell lines

Cell line source(s)	Ramos (ATCC #CRL-1596), Raji (ATCC #CCL-86), CT-26 (Sherie Morrison, UCLA)
Authentication	No cell lines were authenticated.
Mycoplasma contamination	Cell lines were not tested for Mycoplasma contamination.
Commonly misidentified lines (See ICLAC register)	None

Animals and other organisms

Policy information about studies involving animals; ARRIVE guidelines recommended for reporting animal research

Laboratory animals	NSG mice (NOD.Cg-Prkdcscid IL2rgtm1Wjl/SzJ, The Jackson Laboratory # 005557); female; 6-week old
Wild animals	The study did not involve wild animals
Field-collected samples	This study did not involve samples collected from the field
Ethics oversight	Institutional Animal Care and Use Committee

Note that full information on the approval of the study protocol must also be provided in the manuscript.

Flow Cytometry

Plots

Confirm that:

☒ The axis labels state the marker and fluorochrome used (e.g. CD4-FITC).

☒ The axis scales are clearly visible. Include numbers along axes only for bottom left plot of group (a 'group' is an analysis of identical markers).

☒ All plots are contour plots with outliers or pseudocolor plots.

☒ A numerical value for number of cells or percentage (with statistics) is provided.

Methodology

Sample preparation	Retro-orbital bleeds were collected in EDTA-K tubes. Approximately 50ul blood was transferred to 5ml FACS tubes. Erythrocytes were lysed in Ammonium chloride / Sodium Bicarbonate lysis buffer for 10 minutes at room temperature, pelleted and washed in FACS Buffer (0.5% BSA/ 0.01 % Na Azide/4mM Na EDTA). Cells were blocked with normal mouse serum (1:100) for 5 minutes and stained with a cocktail of antibodies containing Anti-Human CD3 APC (Clone OKT3) 1:20, biotinylated Goat F(ab')2 anti-Human F(ab')2 1:100 and 7AAD 1:20. Cells were stained for 30 minutes, washed with cold FACS buffer and stained with Streptavidin PE (1:100) for 30 minutes. Cells were washed a final time and acquired.
Instrument	BD FACSCALIBUR 342795 with Red and Blue Laser
Software	CellQuest v5.2.1 was used for acquisition and analysis was performed with FlowJoV7.6.
Cell population abundance	Relative abundance of the cell type of interest varied depending upon the timing in the study as human T cells undergo an initial expansion in mice followed by a contraction in their peripheral numbers.
Gating strategy	Lymphocyte sized events were gated using FSC and SSC parameters, followed by 7AAD negative live cell events. CD3 positive events were gated using a box gates and were discrete from negative events. Human F(ab')2 PE box gates were set using Fab negative groups for comparison.

☒ Tick this box to confirm that a figure exemplifying the gating strategy is provided in the Supplementary Information.
